# Genome Sequence of *Klebsiella pneumoniae* Respiratory Isolate IA565

**DOI:** 10.1128/genomeA.00896-14

**Published:** 2014-09-11

**Authors:** Jeremiah G. Johnson, Rachel R. Spurbeck, Sukhinder K. Sandhu, Jyl S. Matson

**Affiliations:** a Department of Microbiology and Immunology, University of Michigan Medical School, Ann Arbor, Michigan, USA; b Swift Biosciences, Inc., Ann Arbor, Michigan, USA; c Department of Medical Microbiology and Immunology, University of Toledo, Toledo, Ohio, USA

## Abstract

*Klebsiella pneumoniae* is a clinically significant opportunistic bacterial pathogen as well as a normal member of the human microbiota. *K. pneumoniae* strain IA565 was isolated from a tracheal aspirate at the University of Iowa Hospitals and Clinics. Here, we present the genome sequence of *K. pneumoniae* IA565.

## GENOME ANNOUNCEMENT

*Klebsiella pneumoniae* is a capsulated Gram-negative bacterium that causes a wide range of human infections, including urinary tract infections and severe pneumonia. Even with appropriate treatment, pneumonia caused by this nosocomial pathogen results in a greater than 30% mortality rate in the immunocompetent population, with considerably higher rates in the immunocompromised (1). In order to colonize the human respiratory tract and cause disease, bacteria must adhere to epithelial surfaces to avoid clearance from the respiratory tract. *K. pneumoniae* produces two fimbrial adhesins, type 1 and type 3, which are involved in attachment and biofilm formation in both *in vitro* and *in vivo* models, and are predicted to be virulence factors in human infection (2).

*K. pneumoniae* strain IA565 is a clinical tracheal aspirate isolate of the K15 serotype originally obtained from the University of Iowa Hospitals and Clinics Special Microbiology Laboratory (3). The adherence properties and virulence of this strain have been previously investigated. IA565 is unusual in that it contains two copies of the type 3 fimbrial gene cluster, one copy encoded on the chromosome and one on a large plasmid (3). Interestingly, only the plasmid-encoded MrkD adhesin can bind the type 3 fimbrial substrate, type V collagen, which is present in human extracellular matrix (HECM) and allows IA565 to adhere to trypsinized human buccal cells (3). Recently, the amino acid residues of MrkD that facilitate adherence to type V collagen have been identified, as has the crystal structure of the MrkD HECM binding domain (4).

IA565 is nonpathogenic in a murine model of acute pneumonia (5). Following intratracheal inoculation, IA565 was rapidly cleared from the lungs with no observed morbidity. However, upon intranasal inoculation or oral gavage, this strain was found to stably colonize either the nasal passages or gastrointestinal tract of mice, respectively, for up to 3 weeks (6). Therefore, it is likely that IA565 is a commensal strain of *K. pneumoniae* capable of colonizing murine, and likely human, mucosal surfaces.

*K. pneumoniae* IA565 was grown overnight at 37°C on LB agar, and genomic DNA was isolated using the Qiagen DNeasy blood and tissue kit (Qiagen, Valencia, CA). Genomic DNA was fragmented to an average size of 550 bp using a Covaris M220 (Covaris, Woburn, MA). Fragments were subsequently size selected by Pippin Prep (Sage Science, Beverly, MA), and whole-genome libraries were made using the Accel-NGS 2S DNA library kit (Swift Biosciences, Ann Arbor, MI). These libraries were sequenced on the Illumina MiSeq (Illumina, San Diego, CA) using MiSeq reagent kit v2. Approximately 4 million reads were used for *de novo* genome assembly using MIRA 4 (7), which resulted in 3,922,059 reads assembled into 97 contigs with a total consensus of 5.3 MB (average total coverage of 92×, largest contig of 901 kb and *N*_50_ of 351 kb). A BLAST search confirmed that 55 contigs map to the chromosome and 42 are plasmid contigs.

### Nucleotide sequence accession numbers.

This whole-genome shotgun project has been deposited at DDBJ/EMBL/GenBank under the accession no. JPIQ00000000. The version described in this paper is version JPIQ01000000.
